# The village/commune safety policy and HIV prevention efforts among key affected populations in Cambodia: finding a balance

**DOI:** 10.1186/1477-7517-9-31

**Published:** 2012-07-09

**Authors:** Nick Thomson, Supheap Leang, Kannarath Chheng, Amy Weissman, Graham Shaw, Nick Crofts

**Affiliations:** 1 National Institute for Public Health, Phnom Penh, Cambodia; 2 Nossal Institute for Global Health, University of Melbourne, Melbourne, Australia; 3 Centre for Law Enforcement and Public Health, Melbourne, Australia; 4 Johns Hopkins Bloomberg School of Public Health, Baltimore, USA; 5 World Health Organization, Cambodian Country Office, Phnom Penh, Cambodia; 6 FHI 360, Cambodian Country Office, Phnom Penh, Cambodia; 7 Melbourne School of Population Health, University of Melbourne, 207 Bouverie Street, Parkville, 3100, Australia

## Abstract

The Village/Commune Safety Policy was launched by the Ministry of Interior of the Kingdom of Cambodia in 2010 and, due to a priority focus on “cleaning the streets”, has created difficulties for HIV prevention programs attempting to implement programs that work with key affected populations including female sex workers and people who inject drugs. The implementation of the policy has forced HIV program implementers, the UN and various government counterparts to explore and develop collaborative ways of delivering HIV prevention services within this difficult environment. The following case study explores some of these efforts and highlights the promising development of a Police Community Partnership Initiative that it is hoped will find a meaningful balance between the Village/Commune Safety Policy and HIV prevention efforts with key affected populations in Cambodia.

## 

On the 16th of August 2010, the Co-minister of the Ministry of Interior announced the Village/Commune Safety Policy as a priority policy for the Kingdom of Cambodia. The policy urged authorities at the commune level to ensure that there was no stealing, drug production or dealing, prostitution, child trafficking, domestic violence, gangsters, illegal gaming, use of illegal weapons or crime occurring at any commune in Cambodia [1].

With regards illicit drugs the policy stated that authorities were to specifically:

*“Take action to cut off and eliminate production, dealing and use of illegal drugs at the village commune/Sangkat by following the guideline No 052 National Department of the Police dated 21st 2006 on the implementation of a warlike approach to fighting any drug crime and to especially focus the law on drug control*[1]*.”*

The policy has had negative implications for HIV prevention among risk groups including harm reduction programs; a fact not lost on the Government of Cambodia who have acknowledged that the policy has made it much more difficult to access people who use drugs and has made “service provision for their benefit very difficult due to the misunderstanding of the law enforcement officers, especially at the commune level [2]”.

Although the Village/Commune Safety policy has further highlighted the direct impact that law enforcement policy and practices can have on HIV prevention, many agencies have actively been trying to find a balance between law enforcement and HIV prevention efforts among most-at-risk populations (MARPs) in Cambodia over the last several years. Recognising that law enforcement practices were negatively affecting HIV prevention among sex workers, men who have sex with men and drug users, UNAIDS began working with the National AIDS Authority on a MARPs Community Partnership Initiative (MCPI). The MCPI was being designed as a standard operating protocol that could be implemented to decrease the impacts of law enforcement practices in HIV risk environments. Specifically the MCPI was designed to,

*Restore the enabling environment so as to allow the effective and smooth delivery of all forms of services to all MARPs” –* UN Official familiar with the MCPI

Under the MCPI and with support from USAID and the Global Fund to Fight AIDS, Tuberculosis and Malaria, Family Health International in Cambodia (FHI360) has been piloting initiatives aimed at improving the enabling environments for HIV prevention among MARPs in Banteay Meanchey province. These pilot endeavours have been done in conjunction with the AIDS Secretariat of the Ministry of Interior and focus on strengthening a collaborative partnership among Provincial AIDS Committees/Secretariats, local authorities, police, military police, development partners, non-governmental organisations and members of the MARPs communities. During implementation of this pilot project the local authorities, health care providers and NGO staff conducted sensitisation information sessions to the police about the HIV prevention needs of MARPs and the role of the police in the HIV prevention enabling environment [3].

The police were made aware of the HIV programs working with these groups including the specific activities being implemented to support them. Early evaluations of these sensitisation efforts indicate that the police no longer use possession of condoms by sex workers as evidence for arrest and the police are discussing drug related arrests more carefully with other service providers resulting in them distinguishing drug dealers from drug users and then releasing drug users back to the community [3].

These initial successes are building momentum and FHI360 is being funded by AusAID’s HIV/AIDS Asia Regional Project (HAARP) to conduct a similar pilot program with police in Phnom Penh. Furthermore, officials from the Ministry of Interior involved with the work in Banteay Meanchey are reportedly looking to expand the effort into other provinces. Furthermore, UNODC are also working closely with its partners in Banteay Meanchey and other provinces to ensure that law enforcement officials are working collaboratively with other sectors to enhance the HIV prevention efforts among drug users.

*“There are key officials from the Ministry of Interior who have been providing local and national political support to these pilot program efforts and this is leading officials to want to expand these efforts into Phnom Penh and Sihanoukville.” –* UN Official familiar with the pilot projects.

While the original efforts towards the development of a formal standard operating protocol for the MCPI were with the NAA, it became clear that ultimately the Ministry of Interior needed to be able to lead efforts that were being designed to work with law enforcement officials at the local level, especially in relation to HIV prevention among people who use drugs. The Ministry of Interior agrees and has now specifically requested that resources be redirected so that they can train and build the capacity of all of its law enforcement agencies to play a collaborative leadership role in HIV prevention among MARPs.

*“The Ministry of Interior are key players if we want to make a meaningful impact on the enabling environment for HIV prevention among drug users. There are currently renewed efforts being made to bring the Ministry of Interior further on board with HIV prevention efforts. We need to support these efforts with resources so that the Ministry of Interior can coordinate positive law enforcement efforts with the enabling environment for HIV prevention among MARPs.” –* UN official familiar with the increasing involvement of the Ministry of Interior in HIV prevention

In addition to work on the enabling environment on the ground, efforts are also being made to reform police education with regards harm reduction. With support from HAARP, FHI360 has developed and begun to implement a harm reduction training curriculum for the Cambodian Police training academy. In November of 2011, the National Harm Reduction Curriculum was officially approved by the Cambodian Deputy Prime Minister and early evaluation has demonstrated positive results.

· Training pre-post test results showed a 34 % increase (at least 27 out of 33) in answers provided that favoured a law enforcement approach and support of HR programs.

· A comprehensive nine module curriculum on HIV, drug use and its link with HIV, harm reduction principles and practices and on the new drug laws is being adopted by six police academies around Cambodia [4].

The MCPI project work being done in Banteay Meanchey is about to be reviewed by a delegation that includes the Ministry of Interior. With ongoing technical support from UNAIDS, UNODC, WHO, and FHI360 and in collaboration with the Ministry of Interior the lessons being learned in Banteay Meanchey are being used to develop a new Police Community Partnership Initiative protocol. The protocol may include the formation of Rapid Response Teams to be comprised of police, local authority, NGO representatives and other key stakeholders at the local level to refer MARPs away from police arrest and into HIV prevention services.

It is clear that the Ministry of Interior in Cambodia is serious about improving the role of law enforcement in HIV prevention among MARPs groups and the development and implementation of the Police Community Partnership Initiative is a critical step towards the realization of this goal.

## Competing interests

The authors declare they have no competing interests.

## Authors’ contribution

NT, KC, SL and NC discussed the initial concept of this case study presentation. KC and SL conducted background research into the issue of the village/commune safety policy in Cambodia. NT conducted interviews with key informants familiar with the issue in Cambodia and drafted the initial manuscript. GS and AW provided unique insights, guidance and information with regards to this issue in Cambodia. GS, AW and NC provided editing assistance to reach the final stage of this manuscript. All authors read and approved the final version of this manuscript.
